# Two-Phase MDCT Protocol for the Screening of Small Hepatocellular Carcinoma

**DOI:** 10.3390/jcm11154282

**Published:** 2022-07-22

**Authors:** Anita Paisant, Jérôme Boursier, Djamel Dabli, Jérôme Lebigot, Frédéric Oberti, Sophie Michalak, Valérie Vilgrain, Christophe Aubé

**Affiliations:** 1 Département de Radiologie, Centre Hospitalier Universitaire d’Angers, 49933 Angers, France; dablidjamel@yahoo.fr (D.D.); jelebigot@chu-angers.fr (J.L.); chaube@chu-angers.fr (C.A.); 2 Laboratoire HIFIH, EA 3859, Université d’Angers, 49045 Angers, France; jeboursier@chu-angers.fr (J.B.); froberti@chu-angers.fr (F.O.); somichalak-provost@chu-angers.fr (S.M.); 3 Service de Gastroenterologie et Hépatologie, Centre Hospitalier Universitaire d’Angers, 49933 Angers, France; 4 Département d’Anatomopatologie, Centre Hospitalier Universitaire d’Angers, Université d’Angers, 49933 Angers, France; 5 Département de Radiologie, Hôpital Beaujon, Hôpitaux Paris Nord Val de Seine (AP-HP), 92110 Clichy, France; valerie.vilgrain@aphp.fr; 6 Centre de Recherche sur l’Inflammation (CRI), U1149, Université Paris Diderot, Sorbonne Paris Cité, 75890 Paris, France

**Keywords:** liver neoplasm, screening, irradiation, cirrhosis, computed tomography

## Abstract

Screening programmes for cirrhotic patients are based on ultrasound (US) examinations at 6-month intervals, but a US sensitivity of 47% has recently been reported. The aim of this study was to evaluate a two-phase MDCT protocol in terms of hepatic nodule detection within a hepatocellular carcinoma (HCC) screening situation and to evaluate a reduction in irradiation dose for the 6-monthly checks compared to the classic four-phase protocol. In total, 373 patients with 498 nodules that were suspected to be HCC and ranged from 10 to 30 mm in size were prospectively included. All patients underwent four-phase MDCT with an unenhanced phase, arterial phase (AP), portal phase (PP) and delayed phase (DP). The cumulative irradiation from the repeated 6-monthly MDCT protocol was calculated. Of the 498 nodules, only 4 (0.008%) were only seen in the PP and not in the AP or AP. Of the 319 HCC nodules, 270 (84.6%) had AP hyperenhancement, while 115 had washout in the PP and 224 had washout in the DP. Overall, 222 of the 224 (99.1%) HCC nodules with typical features were seen in the AP and DP. The dose reduction was estimated at 55.4% when using the two-phase protocol (AP and DP). The cumulative irradiation of the two-phase protocol, which was performed every 6 months over 5 years, was 96.5 mSv. MDCT with the two-phase protocol could offer an alternative to ultrasound screening with an interesting risk–benefit trade-off.

## 1. Introduction

Primary liver cancer, mainly hepatocellular carcinoma (HCC), is the sixth most commonly diagnosed cancer and the fourth leading cause of cancer-related deaths worldwide. The World Health Organization predicts that its incidence rate increases annually by about 2% to 4% [1,2]. To allow for curative treatment, HCC must be detected as early as possible. Therefore, screening programmes for cirrhotic patients are based on regular ultrasound (US) examinations at 6-month intervals. However, US is limited by poor sensitivity, which has recently been reported as 47% for early-stage HCC [3]. The increase in the prevalence of obesity has added to the limitations of ultrasound examinations, which is a trend that is set to continue in future. As a consequence, surveillance imaging practices often deviate from practice guidelines, which all recommend only using US. In a study by Joshi et al., only 36% of gastroenterology and hepatology providers in the USA used US exclusively and 60% used two or more imaging modalities [4]. Magnetic resonance imaging (MRI) is the best examination method to screen for HCC in cirrhotic patients [5], but it is too costly to be used in screening programmes and is not widely available. Multidetector CT (MDCT) does not have these same disadvantages and, therefore, presents an alternative. Studies have shown that MDCT is a more effective method for detecting de novo HCC compared to US [6,7,8]. However, the limitation of MDCT is obviously its irradiation, especially when it is carried out every 6 months. In addition, when it comes to CT and cirrhosis, all international guidelines call for CT scans for all four phases (unenhanced phase (UN), late arterial phase (AP), portal phase (PP) and delayed phase (DP)) [9]. The need for CT scans for four phases naturally increases irradiation to an even greater extent. However, the four-phase scan protocol that is presented in all of the international recommendations is intended for diagnosis rather than screening. It is important to note that there is no dedicated protocol for HCC screening using CT scans. To move forwards with MDCT protocols, there is a need to differentiate between screening and diagnosis. The aim of a screening protocol is to detect all of the nodules that are visible on an MDCT, but with a reduced number of phases to make this protocol repeatable. Once nodules are detected during the screening programme, the diagnostic protocol that is recommended in the international guidelines should then be followed.

The aim of this study was to evaluate the ability of a two-phase MDCT protocol to detect nodules in cirrhosis within a large cohort, to evaluate the irradiation dose reduction and discuss the benefits and risks of the irradiation.

## 2. Materials and Methods

### 2.1. Patients

This study was an ancillary study that was based on the database of a large prospective multicentric project [10]. The initial study was approved by the National Ethics Committee and registered under NCT00848952. All patients provided written and informed consent. This study was supported by a national institutional grant.

The database included 442 patients, who were enrolled in 16 expert centres. The inclusion criteria were that patients were followed up for Child–Pugh A or B cirrhosis and had up to three focal liver lesions that were between 10 and 30 mm in diameter, which were identified by US, MDCT or MRI. Patients with a history of HCC that had been treated by surgery or tumour ablation could also be included, but only new nodules that were at a distance of >2 cm from the previously treated lesions were considered. The exclusion criteria were patients with a history of chemoembolisation or a contraindication to contrast-enhanced MDCT or MRI and patients with more than three lesions or with lesions that were larger than 3 cm.

For this ancillary study, all patients with a complete set of MDCT scans in the database (UN phase, late AP, PP and DP) were retrospectively analysed. There were 373 of these patients.

### 2.2. Imaging Protocols

All MDCT examinations were performed using 16 or 64 MDCT scanners. They included unenhanced acquisition, followed by three contrast-enhanced acquisitions (1.5 to 2 mL/kg of iodinated contrast media via IV injection (350 g/L iodine concentration) using a power injector at a rate of 3–4 mL/s, without exceeding 150 mL) during the late hepatic arterial (30 to 35 s), portal venous (70 to 90 s) and delayed (3 min) phases.

Each MDCT scan was analysed on site by a radiologist using all of the tools that were available at their workstation (just as they would in normal clinical practice) and with no knowledge of the other examinations that had been performed or the final diagnoses. The nodule characteristics and enhancement in the different vascular phases (arterial, portal and delayed) were noted. The AP hyperenhancement (APHE) was defined as a non-rim-like enhancement in the arterial phase, which was unequivocally greater (in total or in part) than the liver and higher in attenuation or intensity than the liver in the arterial phase. Hypodensity in the PP or DP was defined as a non-peripheral, visually assessed temporal reduction in enhancement in the whole or part of the liver. “Washout” was defined as hyperenhancement in the AP, followed by hypodensity in the PP or DP.

### 2.3. Final Diagnosis

In total, 262 nodules were histologically proven. The algorithm that was used for final diagnosis has been already described and validated in previous publications [10,11].

### 2.4. X-ray Dosimetric Evaluation

To evaluate the mean X-ray dose of the different MDCT phase combinations, a retrospective analysis was performed on four-phase MDCT examinations. For this, 30 MDCT scans of included patients from one centre were selected at random. The MDCT system that was used was an Aquilion Prime 80 MDCT (Canon Medical System, Otawara, Japan), which used a 3D modulation tube current (sureExposure3D) that modulated the tube current according to the size of the patient, the anatomical region and the acquisition and reconstruction parameters in order to maintain a sufficient signal-to-noise ratio and uniform image quality. The images were reconstructed using AIDR3D (adaptive iterative dose reduction using 3D processing), which is an iterative reconstruction algorithm that allows for reductions in image noise at low doses. The acquisitions were performed at a tube voltage of 120 kV for patients of a typical size. This value was reduced to 100 kV for smaller patients and increased to 140 kV for larger patients. The collimation was set to 80 × 0.5 mm and the reconstructed slice thickness was set to 2 mm.

The dose length product (DLP) value, which was expressed in mGy·cm, was recorded and converted into the effective dose for each of the four acquisitions. Conversion factor values that were based on the ICRP (International Commission on Radiological Protection) publication number 103 were adopted in our study (0.022 mSv/mGy·cm^−1^ for abdomen scans and 0.018 mSv/mGy·cm^−1^ for abdomen/pelvis scans) [12,13].

The total effective dose of the scan was calculated for each examination. The results of this analysis were used to evaluate the effective dose that was associated with each combination of CT phases and estimate the dosimetric savings.

### 2.5. Statistical Analysis

Quantitative variables were expressed as the mean +/− standard deviation (SD). Qualitative variables were expressed as percentages. The sensitivity, specificity, negative predictive value (NPV), positive predictive value (PPV) and diagnostic accuracy were calculated. Then, the sensitivity and specificity were compared using McNemar’s test. Statistical analyses were carried out using SPSS v. 15.0 software (IBM, Armonk, NY, USA).

## 3. Results

The characteristics of the patients are described in Table 1. The 373 patients were made up of 299 men (80.2%) and 74 women (19.8%), with a mean age of 61.4 ± 9.7 years. There were 498 identified nodules, including 319 HCC and 179 non-HCC nodules. Of these, 383 (76.9%) were hyperenhanced in the arterial phase. Of the 115 remaining nodules, 66 were hypoenhanced in the arterial phase and 49 were isodense. Of these 49 nodules, 37 were hypo/hyperdense (34/3) in the DP and 12 were isodense. Finally, of these last 12 nodules, 8 were isodense in the PP, 3 were hyperdense and 1 was hypodense. This meant that only 4 nodules (0.008%) were only seen in the PP and not in the AP or DP.

Overall, 270 (84.6%) of the HCC nodules had AP hyperenhancement. Of these, washout was present in 115 nodules in the PP and in 224 nodules in the DP. All of the HCC nodules with washout in the PP had washout in the DP, except for two. As such, 99.1% of HCC nodules with typical features were seen in the AP and DP.

The diagnostic performances of an AP and PP only protocol and an AP and DP only protocol were compared to the four-phase protocol (Table 2). The sensitivity of the AP and DP protocol was not statistically different from that of the four-phase protocol (70.8% versus 70.2%, respectively (*p* = 0.500)). The specificity of the AP and DP protocol was similar to that of the four-phase protocol (79.3% versus 79.3%, respectively (*p* = 1.000)), as shown in Table 2.

The estimations of the X-ray doses of each MDCT phase and the different MDCT phase combinations are reported in Table 3. The median total effective dose for the classic four-phase MDCT protocol was 21.6 mSv, whereas it was 9.64 mSv for the two-phase protocol with AP and DP, meaning that the dose reduction was 55.4%. Thus, the cumulative irradiation of the two-phase protocol (when performed every 6 months over 5 years) was 96.5 mSv compared to 216.0 mSv for the classic four-phase MDCT protocol.

## 4. Discussion

HCC management has changed over recent years and the limitations of the use of ultrasound in screening programmes have become obvious, which is likely due, in part, to the increasing prevalence of metabolic syndrome [3] and the need to detect small HCC nodules to ensure early treatment. In addition, the current accessibility of MDCT, its precision (which exceeds the technical limitations of ultrasound [6]) and the possibility to view and re-read images afterwards make this type of scan an increasingly attractive option for screening. The current guidelines recommend using ultrasound as an initial screening tool, followed by MDCT or MRI scans for diagnosis and staging. A study by Yoon et al. suggested that biannual two-phase low-dose CT scans have significantly higher sensitivity and specificity values than biannual US scans [8]. Our study showed that 99.99% of the nodules that were visible on the CT scans in the cases of cirrhosis were seen using acquisitions from only two phases: the arterial phase and the delayed phase. This two-phase MDCT protocol for liver exploration could decrease irradiation by 55.4%, which was a significant decrease compared to the classic four-phase MDCT protocol. Moreover, the diagnostic performance was very high, with 99.1% of the HCC features that were identified with the typical four-phase MDCT protocol (APHE and washout) retaining these characteristics in the two-phase MDCT protocol. For a screening process that aims to go beyond the limits of ultrasound, a two-phase MDCT protocol that only includes the arterial and delayed phases should be considered by the guidelines for the screening of cirrhotic patients. It should also be noted that the PP remains necessary for exploring the abdominal cavity as part of the staging process when HCC is discovered during the screening process.

The accuracy of the AP and PP protocol was much lower than that of the AP and DP protocol. This finding was in agreement with older studies that demonstrated the superiority of the DP over the PP for the diagnosis of HCC, which led to the recommendation of the four-phase CT scan protocol for the diagnosis of HCC. Kim et al. showed that the combination of the AP and DP diagnosed more HCC cases than the combination of the AP and PP (75 versus 70 out of 85 HCC cases, respectively) [14]. In a study that included 131 HCC cases, Lim et al. showed that the addition of the DP was helpful for the characterisation of hepatocellular carcinoma in 14% of patients [15]. Cereser et al. showed that washout was present in 44% of cases at PP and in 82% of cases at DP using MRI on a small population (35 patients with 55 HCC nodules) [16]. The main hypothesis to explain the better results of the DP in terms of showing washout is that the arterial supply of well-differentiated HCC nodules is still not exclusive and not sufficient to induce an early washout in the portal venous phase. In line with this hypothesis, Ohashi et al. showed that well-differentiated HCC nodules are not hyperenhanced on MDCT images from the arterial phase [17].

Studies in the literature have shown the superiority of CT over ultrasound in certain screening situations [6,8]. In patients that had been previously treated for HCC, an article by Giangregorio et al. showed that the sensitivity of conventional US was much lower than that of MDCT for the detection of new HCC nodules [18]. Another possibility is for MDCT to be used alternately with ultrasound every 6 months. Indeed, a study by Kim et al. showed that in cirrhotic patients with chronic hepatitis B, alternating between dynamic CT exams and US led to higher detection rates of very early-stage HCC and improved overall survival compared to the use of US exams alone [19]. In this way, CT and MRI have already been integrated in the Japanese Society of Hepatology 2014 guidelines for super-high-risk patients, with the recommendation that the scans are performed every 6–12 months [20].

Irradiation remains a concern for the use of MDCT. The cumulative irradiation for the two-phase MDCT protocol, when performed every 6 months for 5 years, was 96.5 mSv among an adult population compared to 216.0 mSv for the classic four-phase MDCT protocol. The optimisation of the machine parameters for screening programmes could allow this dose to be reduced even further. According to an article by Verdun et al., assuming the natural risk of dying from cancer to be 25%, the additional risk that is associated with an acute exposure of 100 mSv is 1% over a lifetime [21]. According to the so-called “linear no-threshold” theory, the risk that is associated with any exposure is never zero and the risk of inducing cancer increases by approximately 0.5% for an effective dose of 100 mSv [13,22] (which would be reached after 5 years of scans at 6-month intervals with the two-phase MDCT protocol), whereas the risk of HCC in patients with hepatitis C virus and cirrhosis rises to 30% in Japan for a cumulative 5-year incidence. Therefore, the risk–benefit balance would favour the use of MDCT for the earlier detection of small HCC nodules.

The repeated injection of iodinated contrast agent should not be forgotten and must also be taken into consideration. Indeed, this proposed screening strategy could not be applied to patients who are at risk of post-contrast acute kidney injury. It is all about balancing the benefits and risks of early cancer (HCC) detection and kidney risk factors, respectively. Some strategies can be systematically employed to reduce kidney risks, such as the systematic use of isotonic contrast agents, an optimal volume and injection protocol and hyperhydration when performed at home by the patient before examination. In view of the benefits and risks, this screening strategy could initially be proposed to Child–Pugh A patients as there was an inverse correlation between the glomerular filtration rate and Child–Pugh score [23].

Finally, the health economic point of view must also be taken into consideration. Unfortunately, this study was not designed as an evaluation of health economics, so the following assumptions were based on previous studies in the literature. A systematic review by Nguyen et al. regarding the evaluation of health economics for hepatocellular carcinoma screening strategies confirmed that CT and MRI are clinically more effective than US when performed at the same intervals, but they are much more costly [24]. Lima et al. compared imaging-based surveillance and diagnostic strategies for patients who were at risk of hepatocellular carcinoma (HCC) while taking into account technically inadequate examinations and patient compliance [25]. Their conclusion was that the use of CT for HCC surveillance and diagnosis and a complete MRI to compensate for inadequate CT scans was the most cost-effective strategy. Abbreviated MRI protocols have also undergone health economic evaluation and have been found to be cost-effective for HCC screening among at-risk populations in comparison to US [26]. Nevertheless, in clinical practice, many patients with low echogenicity already benefit from CT follow-up scans (e.g., 50% of patients in a study by Joshi [4]). A long-term health economics study is needed to explore the financial impacts of the use of MDCT versus the benefits of the earlier discovery and management of HCC in cirrhosis patients.

This study had some limitations. Firstly, the histological gold standard was not available for all of the patients; nevertheless, we had a large proportion of nodules with histological evidence (262 nodules). However, a histological reference for all nodules is no longer recommended and would not be ethical. In addition, biopsies (which are recommended when diagnosis cannot be achieved using non-invasive means) were mainly used to diagnose the cases of atypical nodules. Therefore, to ensure a realistic proportion of typical and atypical HCC nodules, we included nodules that had no histological references. We used a step-by-step algorithm to provide the final diagnosis of these nodules to ensure the reliable gold standard that has already been validated in the literature [10,11]. Secondly, the dose was evaluated using examinations from a single centre. As the collection of these dose data was retrospective, some centres were no longer able to provide us with this information due to a change in their PACS. Nevertheless, this centre used “optimal +” technical parameters, which corresponded to a level of irradiation that was within a higher dosage range than is usually used in most centres for clinical practice. Therefore, we could consider these to be maximised dose results and the final cumulative dose over 5 years could even be lower than that proposed in the study. In addition, the development of new generations of MDCT with lower doses and similar image performances could reduce doses even more in the future and strengthen the acceptability of screening with MDCT. Thirdly, our results were not directly compared to screening with US. However, some studies have already shown that MDCT is a more effective method than US for the detection of de novo HCC [6,7,8] and our goal was to further investigate the feasibility of MDCT screening protocols.

## 5. Conclusions

MDCT with a two-phase protocol with its reduced irradiation level offers an alternative to ultrasound screening that has an interesting risk–benefit trade-off.

## Figures and Tables

**Table 1:** The characteristics of the patients.

Gender	
Men (%) Women (%)	299 (80.2) 74 (19.8)
Age (Mean +/− SD)	61.4 +/− 9.7
BMI kg/m² (Mean +/− SD)	26.7 +/− 4.8
Presence of Cirrhosis (%)	365 (97.9 ^†^)
Child–Pugh Score (%) *	
A	265 (71.0)
B + C	88 (25.6)
Aetiology of Liver Disease ^‡^	
Alcohol (%) Hepatitis B (%) Hepatitis C (%) Metabolic (%) Other (%)	215 (57.6) 34 (9.1) 113 (30.3) 75 (20.1) 34 (9.1)
Patients with Serum AFP Above the Upper Normal Laboratory Limit ^§^ (%)	102 (27.3)
Number of Nodules per Patient	
Patients with One Nodule (%) Patients with Two Nodules (%) Patients with Three Nodules (%)	278 (74.5) 65 (17.4) 30 (8.1)
Size of Nodule (mm; Mean +/− SD)	17.4 +/− 6.2

^†^ Non-cirrhotic patients with chronic liver disease; * 13 had missing data; ^‡^ some patients had several aetiologies; ^§^ 62 had missing data.

**Table 2:** The performance of the different combinations of dynamic phases for the diagnosis of HCC.

	Se (%)	Sp (%)	PPV (%)	NPV (%)	Acc (%)
**MDCT** Four-Phase Protocol	70.8	79.3	85.9	60.4	73.9
**MDCT** AP + PP only	36.1	92.7	89.8	44.9	56.4
*p* *	<0.001	<0.001			
**MDCT** AP + DP only	70.2	79.3	85.8	59.9	73.5
p *	0.500	1.000			

* Compared to the four-phase protocol; Se, sensitivity; Sp, specificity; PPV, positive predictive value; NPV, negative predictive value; Acc, accuracy; AP, arterial phase; PP, portal phase; DP, delayed phase; MDCT, Multidetector CT.

**Table 3:** The X-ray dosimetry according to the four-phase and two-phase MDCT protocols.

Protocol	Acquisition	Median DLP (Q1, Q3) (mGy·cm)	Median Effective Dose (Q1, Q3) (mSv)	Irradiation Decrease
**Classic Four-Phase Protocol**	UN Liver	228 (173, 318)	5.02 (3.8, 7)	
	AP Liver	219 (171, 294)	4.82 (3.7, 6.5)	
	PP Abdomen and Pelvis	384 (292, 463)	6.91 (5.3, 8.3)	
	DP Liver	219 (171, 295)	4.82 (3.7, 5.5)	
	**Total**		**21.60 (16.7, 27.3)**	
**Two-Phase Protocol**	AP Liver	219 (171, 294)	4.82 (3.7, 6.5)	**55.40%**
	DP Liver	219 (171, 295)	4.82 (3.7, 5.5)	
	**Total**		**9.64 (7.4, 13.0)**	

Q1 and Q3, first and third quartiles; UN, unenhanced phase; AP, arterial phase; PP, portal phase; DP, delayed phase.

## Data Availability

The study data can be found in the research department of Angers University Hospital, France.
